# DNA methylation and telomere length in 2–5 year olds with intrauterine preeclampsia exposure: a P4 sub-study

**DOI:** 10.1186/s13148-025-02029-1

**Published:** 2025-11-24

**Authors:** Jason P. Ross, Benjamin J. Varley, Jadon K. Wells, Robyn P. L. Yeh, Hilda A. Pickett, Raja S. Vasireddy, Lynne Roberts, Greg Davis, Amanda Henry, Maria E. Craig, Megan L. Gow

**Affiliations:** 1 Human Health Program, CSIRO Health & Biosecurity, Westmead, NSW Australia; 2 The George Institute for Global Health, Randwick, NSW Australia; 3 Department of Women’s and Children’s Health, St George Hospital, Kogarah, NSW Australia; 4 Telomere Length Regulation Unit, Children’s Medical Research Institute, University of Sydney, Westmead, NSW Australia; 5 Dept of Haematology, Sydney Children’s Hospitals Network (SCHN), Children’s Hospital at Westmead, Westmead, NSW Australia; 6 School of Clinical Medicine, UNSW Medicine and Health, St George and Sutherland Clinical Campus, Kogarah, NSW Australia; 7 Discipline of Women’s Health, School of Clinical Medicine, UNSW Medicine and Health, Kensington, NSW Australia; 8 Discipline of Paediatrics and Child Health, School of Clinical Medicine, UNSW Medicine and Health, Kensington, NSW Australia; 9 The University of Sydney Children’s Hospital Westmead Clinical School, Westmead, NSW Australia

**Keywords:** Preeclampsia, Children, Epigenetics, DNA methylation, Telomere length

## Abstract

Preeclampsia is a hypertensive disorder of pregnancy characterised by new onset hypertension at ≥ 20 weeks’ gestation accompanied by maternal organ dysfunction and/ or fetal compromise. This hypertensive exposure in pregnancy has known long-term consequences for both the mother and child. Presently, the mechanisms for these cardiometabolic, immunological and neurodevelopmental consequences in the child aren’t well characterised, but epigenetic changes in development and premature biological aging may play a role in the development of these long-term morbidities. To investigate, we assessed genome-wide DNA methylation and biological aging in the blood of 2–5 year-old children with (n = 20) or without (n = 20) previous intrauterine exposure to preeclampsia. Exposure to preeclampsia was associated with 103 differentially methylated regions (DMRs) proximal to both known and novel candidate genes. Biological aging, as determined by two telomere length quantification methods and an epigenetic clock, was found to not be statistically different between the preeclampsia exposure and normotensive pregnancy groups. From the 103 DMRs, gene ontology analysis highlighted that 17 regions are proximal to genes involved in cell–cell adhesion (p = 7.49 × 10^–12^), and more specifically homophilic cell adhesion (p = 7.49 × 10^–12^). This is the first study to consider genome-wide epigenetic changes in the blood of young children exposed to preeclampsia in utero, with previous studies concentrating on cord blood or placental biopsy material collected at birth.

**Supplementary Information:**

The online version contains supplementary material available at 10.1186/s13148-025-02029-1.

## Introduction

Preeclampsia is defined by the International Society for the Study of Hypertension in Pregnancy (ISSHP) as hypertension (BP ≥140 mmHg systolic and/or ≥90 mmHg diastolic) associated with the new onset at ≥ 20 weeks’ gestation of at least one of 1) proteinuria, 2) other maternal end-organ dysfunction 3) uteroplacental dysfunction [1]. Preeclampsia affects 2–5% of pregnancies globally [1, 2], is a leading cause of maternal morbidity and mortality [3, 4], and is associated with a range of poor health consequences for the exposed fetus. In the short-term, this includes increased rates of fetal growth restriction, placental abruption, stillbirth and neonatal morbidity and mortality [5, 6]. Beyond birth outcomes, exposure to intrauterine preeclampsia has been associated with cardiometabolic, immunological and neurodevelopmental morbidities [7–13].

Early changes to genome regulation in response to preeclampsia, including DNA methylation and telomere length, may contribute to the precipitation of the long-term morbidities associated with exposure. DNA methylation, essential for mammalian development, is an epigenetic mechanism that chemically modifies DNA, regulates gene activity for cell division and is the mechanism behind parent-of-origin imprinting in the embryo [14]. DNA methylation is erased from the fertilised embryo, with subsequent repatterning during fetal development being highly specific to the stage of fetal development and the various bodily tissues. This process is potentially affected by exposure to intrauterine stressors such as preeclampsia, gestational diabetes, and cigarette smoking [15–17]. In a 2019 study by the Pregnancy and Childhood Epigenetics (PACE) Consortium, preeclampsia was associated with multiple DNA methylation marks in cord blood at birth [18]. Longitudinal analyses from a sub-set of this study demonstrated persistence of these DNA methylation marks at age 7 and 14 years, suggesting that the association of preeclampsia and other hypertensive disorders of pregnancy with differential methylation in cord blood persists beyond birth [18]. Another study in 24 young adults demonstrated that intrauterine exposure to hypertensive pregnancies (sample size: n = 5) led to epigenetic changes compared with normotensive pregnancies (n = 19) [19]. However, these findings may not be generalisable to childhood years and preeclampsia specifically.

The length of telomeres, DNA–protein structures that serve to protect the chromosome ends, are emerging biomarkers of oxidative stress and have been related to various age-related diseases [20]. While they naturally shorten with advancing age, short telomere lengths in early life indicate accelerated biological ageing influenced by heredity and exposure to environmental impacts [21], including in utero exposure to maternal conditions such as gestational diabetes and increased maternal weight [22, 23]. To date, investigation of the impact of preeclampsia exposure on telomere length has been limited to placental tissue and cord blood, with mixed results. One study (n = 57) reported reduced telomere lengths in placental trophoblasts from women with preeclampsia and/or intrauterine growth restriction via fluorescence in situ hybridisation (FISH) [24], another study (n = 26) also reported shorter placental trophoblast telomeres via FISH [25]. A later study (n = 471) estimated telomere length and mitochondrial DNA (mtDNA) copy number via PCR assays and found longer telomeres in maternal and cord blood DNA from PE cases, higher mtDNA copy number in maternal blood, but lower mtDNA copy number in cord blood [20]. No study has been conducted in early childhood following preeclampsia exposure.

DNA methylome data can also be used to estimate biological age in the form of an epigenetic clock. Biological age is matched with chronological age and the residual variation in epigenetic age independent of chronological age, called epigenetic age acceleration (EAA), is a measure of the speed of an individual’s aging relative to the reference population. In more technical detail, EAA is expressed as the residual from a linear model regressing epigenetic age as the response variable onto chronological age as the predictor variable. EAA has had mixed associations with adverse early life events in paediatric populations [26]. There is known inaccuracy in predicting age in paediatric versus adult populations, presumably as the rate of change of DNA methylation is greater in the paediatric population compared to adulthood [27]. This has motivated the construction of the paediatric and buccal-cell specific, Paediatric-Buccal-Epigenetic (PedBE) clock [28]. Also of utility for paediatric samples, the skin and blood clock outperforms earlier clocks in accuracy [29] on saliva and skin, endothelial, and blood cells and has an adjustment function for samples with a chronical age under 20 years old [29].

Epigenomic mechanisms involved in fetal programming and telomere length may play important roles in mediating the increased risk of cardiometabolic, immunological and neurodevelopmental morbidities apparent in children born following a pregnancy affected by preeclampsia [7–13]. A greater understanding of the impact of preeclampsia on the genomic regulation of children has the potential to lead to the development of therapeutic targets that can be administered before clinical disease manifests [19]. Here, we assessed DNA methylation and biological aging (via telomere length and an epigenetic clock) in children aged 2–5 years of age with or without a previous intrauterine exposure to preeclampsia.

## Methods

### Participant characteristics

This was a cross-sectional sub-study of offspring from participants in the Postpartum Physiology, Psychology and Pediatric follow up study (P4 study), a prospective, observational study of postpartum women with either normal blood pressure (BP) or preeclampsia in their preceding pregnancy, conducted at St George Hospital, Sydney, Australia. A study protocol for the P4 cohort has been published [30].

Eligibility for women in the P4 study included a good understanding of written and spoken English, and birth of a singleton live baby within the previous 6 months. Women were excluded if they were pregnant again at 6 months, if their baby was born with a congenital anomaly, or they had diabetes, chronic hypertension, renal or other serious disease prior to pregnancy. The recruited cohort consisted of 90 women who had preeclampsia during pregnancy and 302 control women who had a normotensive pregnancy. Preeclampsia was defined as persistent de novo hypertension (systolic BP ≥ 140 mmHg and/or diastolic BP ≥ 90 mmHg) that developed at or after 20 weeks’ gestation accompanied with one or more of the following new-onset conditions: proteinuria, other maternal organ dysfunction including liver or kidney involvement, neurological complications, low platelets or uteroplacental dysfunction, according to the ISSHP guidelines [1].

This cross-sectional sub-study was conducted between July 2020 and April 2021. All children born to P4 mothers and aged 2–5 years during this time period were eligible for recruitment. Ethical approval for this sub-study was obtained from the South Eastern Sydney Local Health District Human Research Ethics Committee (2019/ETH11984). Data included in this sub-study were collected when the child was 2–3 years of age. The vascular health outcomes of children participating in this sub-study have been published [31].

Pediatric assessments including height, weight BMI and birth weight Z-scores, and maternal demographics were measured as previously described [31].

### Sample preparation and processing

In total, 20 ml of blood was collected from each participant by blinded personnel. Samples were centrifuged at 2000 × *g* RCF for 10 min, before separating the buffy coat and plasma, and storing at -80° C at St George Hospital. DNA was extracted from the buffy coat using a QIAGEN QIAamp DNA Blood Mini Kit according to the manufacturers protocol. Quality assessment of the DNA extractions was performed by QuantiFluor. Leukocyte telomere length was determined from purified genomic DNA and measured by quantitative PCR and terminal restriction fragment (TRF) length analysis. Leukocyte DNA methylation was examined via the Illumina MethylationEPIC BeadChip (EPIC) microarray at the Australian Genome Research Facility (AGRF), Melbourne, Australia.

The EPIC arrays were processed according to our statistical blocking plan. To avoid confounding with batch effects, the DNA samples were plated onto six glass slides (BeadChip arrays) and allocated to the blocking plan so that both male and female children from both normotensive and preeclamptic pregnancy were represented on each slide.

Individual samples were normalised to approximately 500 ng of DNA in 45 μL and bisulphite converted with the Zymo EZ-96 DNA Methylation kit. Samples were processed on the EPIC BeadChip following the Illumina Infinium HD Methylation Assay. Raw fluorescent signal intensities were extracted using the Illumina iScan system.

### EPIC BeadChip preprocessing

All statistical analysis for the EPIC BeadChips was carried out in R (v4.5.1). In initial quality control, all EPIC arrays were found to have a consistently high detection p-values​ with a mean of only 666 (and a maximum of 1,032) failed probes per array. The quantitative control probes were all consistently at the expected fluorescence levels for high quality staining, target removal, hybridisation, extension, conversion and specificity. A PCA analysis demonstrated no outlier samples.

DNA-methylation predicted sex, as determined by the minfi (v1.54.1) function getSex, also conformed to the recorded sex of the participant. Cell types in the blood were estimated via EpiDISH (v2.24.0) [32] on BMIQ normalised β values [33]. BMIQ normalisation was used for this estimate, as the EpiDISH authors use this normalisation method to generate their blood reference values. The EPIC arrays were also preprocessed using the Dasen procedure [34] from the R package, watermelon (v2.14.0) and noob [35] from the R package, minfi (v1.54.1) [36]. A number of studies have found Dasen to be an optimal between-array preprocessing method, so this was used for downstream analyses [37–39]. After Dasen normalisation, 2,443 β values were reported as missing. These were converted to β of mean = 0.5 with a small amount of random noise (standard deviation = 1 × 10^–8^). This random noise was introduced as the ComBat batch-correction method needs a variance > 0 for each CpG probe. Before conversion to M-values, 5 β of exactly 0 or 1 were shifted by a small offset of 0.005 to avoid infinite M-values.

### Probe filtering

Each CpG site probe was mapped to SNPs from dbSNP v151 [40] and the distance from the CpG site to the nearest documented SNP calculated using the functionality in GenomicRanges (v1.60.0). A list of probes considered as batch-effect prone in three MethylationEPIC reference sets was used to filter out probes prone to technical variation [38]. Probes considered batch-effect susceptible had a log variance ratio < log2(1/1.5) and a mean beta difference > 1% across all three reference datasets.

Discarded were 5,373 probes with a detection p-value > 0.01 in at least one sample, 41,560 probes with SNPs at the CpG site or 1 bp proximal, 19,627 probes on the X or Y chromosomes, 29,233 probes identified by Chen et al. [41] as being cross-reactive, 14,394 probes identified by Ross et al. [38] as being consistently batch-effect prone and 11,057 as erroneously corrected. The number of probes remaining for statistical testing was 764,905 probes.

### Differential methylation analysis

The intended model specification was that methylation at a given CpG site is conditional upon the child’s preeclampsia status, sex, age at blood draw, and their estimated blood cell proportions. As blood cell proportions are compositional data, where the increase in one blood cell results in the lowering of others, there was an expectation of high multicollinearity. To measure the degree of multicollinearity, simple Pearson correlation matrices of the blood cell proportions were generated with the ‘cor’ function in R, and variance inflation factor (VIF) analysis was performed using the R package, car (Companion to Applied Regression, v3.1–3). The VIF provides an index measuring how much the variance of a regression coefficient is increased due to collinearity. One accepted rule of thumb is that a VIF > 5 is a limit for multicollinearity in a model.

Linear regression for differentially methylated probe (DMP) analysis was undertaken using Limma (v3.64.3) [42]. The model was specified as:

$$ \begin{aligned} M_{i} \sim & {\text{ preeclampsia status }} + {\text{ sex}} \\ & \quad + {\text{ age }} + {\mkern 1mu} CD4^{ + } T{\mkern 1mu} + {\mkern 1mu} CD8^{ + } T{\mkern 1mu} + {\mkern 1mu} B{\mkern 1mu} \\ & \quad + {\text{ Natural Killer }} + {\text{ Monocytes}} \\ & \quad + {\text{Eosinophils}} \\ \end{aligned} $$

where M_*i*_ is the M-value of the *i*-th CpG site on the EPIC array. Differentially methylated region (DMR) analysis was undertaken with DMRcate (v3.4.1) [43] using the default settings (lambda = 1000 and C = 2) and the same model specification as above. DMRcate takes results produced by Limma and implements Gaussian kernel smoothing to find CpG sites which are colocalised in their differential methylation across groups. This approach reweights the t-statistic at a given CpG site based on the squared t-statistics of neighbouring CpG sites. This property of sharing of information between nearby CpG sites makes use of regional correlation and can drive down spurious results supported by only one probe. To account for multiple testing, p-values were corrected via the Benjamini–Hochberg false discovery rate (BH-FDR) method and a p-value considered significant when below 0.05.

### Batch correction

In line with recommendations, M-values were used for batch correction [38]. The ComBat method from the library sva (v3.56.0) [44] was used to rerun the same model specification as above and nominating slide identifier (“Sentrix ID”) as the batch variable to correct. Batch-corrected M-values were then used to undertake again the DMP and DMR analysis as above.

### Enrichment analysis

For the DMRs only, enrichment analysis for gene ontology (GO) terms, Kyoto Encyclopedia of Genes and Genomes (KEGG) pathways and 50 Hallmark gene sets from the Molecular Signatures Database (MSigDB) was undertaken via the R package missMethyl (v1.42.0). The missMethyl R library was used as this approach appropriately considers bias resulting from a differing number of probes per gene present on the EPIC array [45].

### DNA methylation clock analysis

The skin and blood epigenetic clock [29] was calculated using the R package methylclock (v1.14.0) [46]. The residuals after regressing chronological age and DNA methylation age, and those after regressing chronological age and DNA methylation adjusted for cell counts were used for later analysis.

### Telomere quantitative PCR analysis

Telomere Quantitative PCR (qPCR) was performed relative to the single copy gene HBG using a two standard curve method described previously [47–49]. The telomere primer sequences [47] were synthesised by IDT (Singapore). The primer sequences were: forward 5’- GGTTTTTGAGGGTGAGGGTGAGGGTGAGGGTGAGGGT-3’, reverse 5’- TCCCGACTATCCCTATCCCTATCCCTATCCCTATCCCTA-3’. HBG primers were: forward 5’-GCTTCTGACACAACTGTGTTCACTAGC-3’, reverse 5’-CACCAACTTCATCCACGTTCACC-3’. Results were presented as relative telomere content (arbitrary units). Experiments were carried out in a Rotor-Gene Q platform (Qiagen, Maryland, United States) and analysed using Rotor-Gene 6000 series software (Qiagen, Maryland, United States).

#### Terminal Restriction Fragment (TRF) length analysis

Terminal restriction fragments (TRFs) were obtained from genomic DNA by complete digestion with the restriction enzymes *Hinf*I and *Rsa*I. TRFs were separated by pulsed-field gel electrophoresis, as described previously [50]. Gels were dried, denatured, and subjected to in-gel hybridization with a γ-[32P]-ATP-labelled (CCCTAA)_4_ oligonucleotide probe. Gels were washed and the telomeric signal visualized by PhosphorImage analysis. Quantitation was performed using ImageQuant TL software.

## Results

Sixty-nine women participating in the P4 Study consented for their child to participate in this study. Of these 69, a sufficient sample of blood was obtained from a total of 20 children exposed to intrauterine preeclampsia and 20 controls for inclusion in this sub-study.

From the 40 families included in this sub-study, children exposed to preeclampsia were born at a younger gestational age (37.7 vs 39.1 weeks, Table 1). There were no other significant differences between groups (Bold in Tables 1 and 2 denotes significance at an alpha of 0.05) . The 20 mothers in the substudy with preeclampsia had higher rates of antihypertensive medication during pregnancy (60% vs 0%), higher rates of caesarean Sect. (65% vs 15%) and were moderately active on more days of the week (median 2 days/week versus 0 days/week; Table 2) compared with normotensive mothers. Mothers with preeclampsia were classified as early vs late onset (15% vs 85%, respectively) and severe hypertension preeclampsia vs non severe (40% vs 60%, respectively; Table 2).

**Table 1:** Offspring characteristics stratified by exposure to preeclampsia vs normotensive pregnancy

	Preeclampsia n = 20	Normotensive n = 20	p-value
Male	12 (60)	13 (65)	0.774
Age at assessment (Years)	4.3 [2.9, 5.1]	5.0 [2.8, 5.2]	0.552
Height (cm)	108.5 [96.7, 112.0]	109.0 [93.6, 111.7]	0.756
Height Z-score	0.56 ± 1.05	0.12 ± 0.81	0.145
Weight (Kg)	17.2 ± 3.5	17.0 ± 3.3	0.854
Weight Z-score	0.39 ± 1.01	0.26 ± 0.85	0.641
BMI (kg/m^2^)	15.6 ± 1.3	15.8 ± 1.2	0.552
BMI Z-score	0.06 ± 0.90	0.24 ± 0.86	0.552
Birth weight (kg)	2.92 ± 0.67	3.24 ± 0.53	0.068
Birth weight Z-score*	-0.32 ± 0.83	-0.22 ± 1.12	0.649
Birth length (cm)	48.7 ± 4.0	49.7 ± 2.7	0.374
Birth length Z-score*	-0.07 ± 1.01	-0.22 ± 1.21	0.680
Gestational age (weeks)	37.7 ± 2.4	39.1 ± 1.4	**0.017**
Preterm birth	6 (30)	2 (10)	0.114
Months breastfed	11.0 ± 7.9	12.9 ± 6.0	0.389

Data presented as n (%), mean ± SD or Median [IQR]

^*^Corrected for gestational age

BMI: Body mass index

**Table 2:** Maternal characteristics stratified by preeclampsia vs normotensive pregnancy

	Preeclampsia	Normotensive	*p*-value
	n = 20	n = 20	
Age at birth (years)	32.5 ± 4.8	32.8 ± 3.8	0.871
Smoking ever at 6 months	7 (35)	6 (30)	0.736
Alcoholic drinks / week at 6 months (drinks)	1 [0, 2]	0 [0, 2]	0.597
Moderate exercise / week at 6 months (days)	2 [1.3, 3]	0 [0, 2]	**0.025**
Illicit drug use ever at 6 months postpartum	5 (25)	11 (55)	0.053
Antihypertensive medication during pregnancy	12 (60)	0 (0)	**< 0.0001**
*Parity:*			
1	15 (75)	10 (50)	0.102
2	5 (25)	10 (50)	
Ethnicity: Oceanian	10 (50)	9 (45)	0.752
Pre-pregnancy BMI (kg/m^2^)*	25.4 ± 4.4	23.7 ± 3.7	0.201
Dating scan weight (kg)	66.6 ± 11.0	63.6 ± 11.3	0.4
Caesarean section	13 (65)	3 (15)	**0.001**
*Education:*			
Tertiary	7 (35)	2 (10)	0.058
University	13 (65)	18 (89)	
GDM/GDM in previous pregnancy	3 (15)	1 (5)	0.292
*Preeclampsia onset:*			
Early	3 (15)	–	–
Late	17 (85)	–	
*Preeclampsia blood pressure severity:*			
^1^Hypertension	8 (40)	–	–
^2^Severe hypertension	12 (60)	–	

Data presented as n (%), mean ± SD or Median [IQR]

Categorical data calculates with Chi square or Fisher’s exact

BMI: Body mass index, DBP: diastolic blood pressure, GDM: Gestational diabetes mellitus. SBP: systolic blood pressure

^*^Based on self-reported weight

^1^Defined as Systolic blood pressure ≥ 140 mmHg and/or diastolic blood pressure ≥ 90 mmHg

^2^Defined as Systolic blood pressure ≥ 160 mmHg and/or diastolic blood pressure ≥ 110 mmHg

### DNA methylation exploratory data analysis

After BeadChip preprocessing, multivariate analysis was used to inspect the methylation data and identify influencing factors. Principal component analysis (PCA) demonstrated that cell composition, age and sex are large sources of biological variation in the data. Slide number (batch effect) was also apparent (Supplemental Fig. 1). Considering the influence of cell composition, this was investigated further. Across the samples, the mean blood cell type estimates generated by EpiDISH suggested the blood was composed of mostly neutrophils (42.58%), CD4^+^ and CD8^+^ T-cells (21.00% and 15.04%, respectively), B-cells (11.80%) and monocytes (6.42%). The levels of eosinophils (mean = 0.94%) and natural killer cells (mean = 2.22%) were highly skewed across this child cohort.

**Fig. 1:**
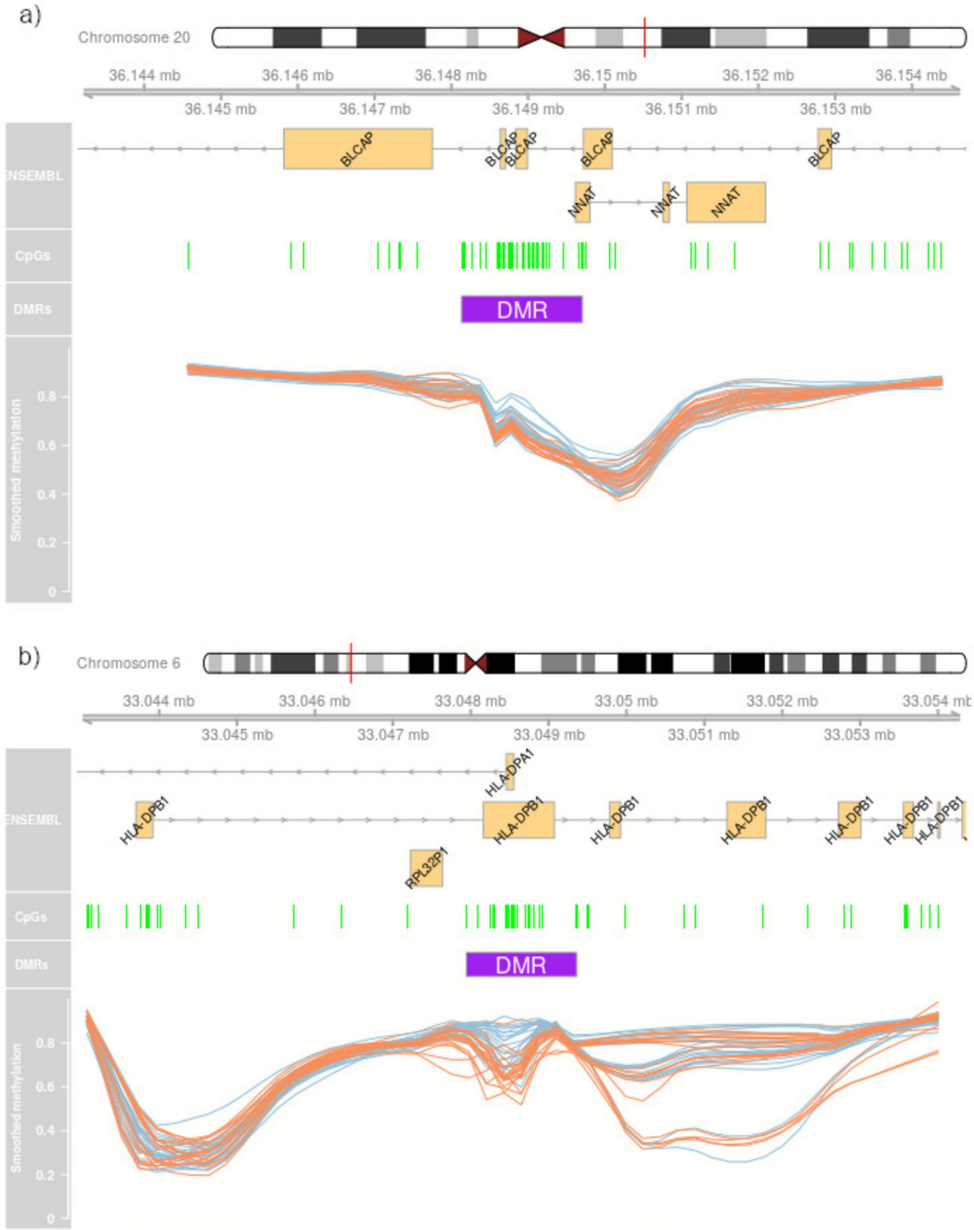
Differentially methylated region (DMR) examples. DNA methylation betas are presented for children exposed to intrauterine preeclampsia (orange) and normotensive pregnancy (blue), with the location of the CpG sites on the microarray also illustrated (green). **a** DMR2, a 1.57 kbp DMR spanning 40 CpG sites, exhibits less DNA methylation and reduced variability in blood from PE-exposed children. It covers two exons of the antisense *BLCAP* locus and is directly upstream in the promotor region of the sense-orientated *NNAT* gene. **b** for DMR4, a 1.42 kbp DMR spanning 12 CpG sites across exons of the antisense *HLA-DPA1* and sense *HLA-DPB1* loci, there is reduced methylation in PE-exposed children accompanied by a triclustered (high, intermediate, low) pattern of methylation just downstream of the DMR which is typical of methylation quality trait loci (mQTL)

### Differentially methylated probe (DMP) analysis

Cell-type heterogeneity is a concerning confound in EWAS studies and in keeping with best practice [51], we included cell-type composition analysis in the linear model. This avoids the potential for false positive significant probes due to differences in age, recent infection with a respiratory virus, hay fever, asthma episodes, or other immune modulating factors across the pregnancy history groups.

As blood cell type estimates are all proportions summing up to 1.0, adding all the cell types into the model is likely to introduce some undesirable multicollinearity. Therefore, a correlation matrix and Variance inflation factor (VIF) scores were used to understand the collinearity. Neutrophil proportions were found to correlate with all other blood cell type proportions except monocytes, and neutrophils, monocytes and natural killer cell proportions had the least correlation with each other. Neutrophils and CD4^+^ T-cell counts were highly correlated (R = -0.709) and are likely overly colinear for a linear regression model. VIF analysis showed that the neutrophil count was particularly collinear and removing it was sufficient to remove undue collinearity (all VIF scores < 5).

The model was specified such that methylation at a given CpG site is conditional upon preeclampsia status with sex, age and blood cell proportions other than neutrophils as covariates (CD4^+^T and CD8^+^T, B and Natural Killer cells, monocytes and eosinophils). CpG sites were filtered as described in the Materials and Methods and a differentially methylated probe (DMP) analysis was performed using Limma on Dasen normalised data. In total, 1,292 FDR-corrected significant DMPs were found for sex and 181 for age, but none were associated with preeclamptic pregnancy. Across the blood cell types, 7,180 to 49,224 DMPs were found for each, except for monocytes, for which only 13 DMPs were associated (Table 3).

**Table 3:** Differentially methylation probes (DMPs) with and without batch-correction

DNA methylation	PE	Sex	Age	CD4 + T	CD8 + T	B	NK	Eosinophil	Monocyte
*Uncorrected data*									
Decrease	0	439	154	16,001	7,487	5,627	14,591	6,018	13
Increase	0	853	27	33,223	18,841	8,551	20,099	1,162	0
*Batch-corrected data*									
Decrease	382	4,327	2,775	35,643	16,562	13,903	36,738	12,633	1,329
Increase	368	8,780	1,468	54,241	32,040	18,456	39,446	5,680	1,045

Exploratory PCA-based unsupervised multivariate analysis found evidence of batch-effect and this technical variance would be detrimentally adding to sample variance. To remove this confounder and look further for associations with preeclampsia, we used the ComBat batch-effect correction approach to remove this technical variance; with the slide identifier specified as the batch covariate, and the same model as above specifying covariates with biological variance to preserve. After batch correction, 750 CpG sites were found to be associated with exposure to preeclampsia (Table 3). The number of DMPs associated with other covariates also increased.

To examine for gender- or age-specific differences we also undertook a subset analysis and split the cohort into male and female subsets and also dichotomised by age (two groups from 2.0 up to 3.7 years and from 3.7 to 5.4 years). For either comparison, no probes passed the genome-wide significance threshold. If the cohort was split by hypertensive severity compared to normotensive pregnancy exposure, some probes crossed the significance threshold (2 DMP for non-severe exposure and 33 DMP for severe exposure). Splitting by preeclampsia onset also returned 544 significant probes for late onset only, but none for early onset only. Supplemental Table S1 presents the overlaps for each comparison.

### Differentially methylated region (DMR) analysis

By design, batch-effect correction reduces false-negative results, but in certain circumstances it can also introduce false positives [52, 53]. To counter for the possibility of spurious associations, we cautiously chose to concentrate on DMRs rather than DMPs. As DNA methylation is known to exhibit regional correlation [54], we assume that genuine (true positive) associations often have neighbouring CpG sites exhibiting the same differential methylation, and this phenomenon can be used to filter out individual spurious (false positive) DMPs. Differentially methylated region analysis was undertaken with DMRcate using the recommended default settings for array data. To control for Type I error, this sets the FDR cutoff (Benjamini-Hochberg) for which CpG sites are individually called as significant to an alpha level of 0.05. For dichotomous phenotypes, DMRcate is known to be conservative and control genome-wide false positives rates appropriately [55]. All steps for preprocessing, batch effect correction, and DMR calling have been uploaded onto GitHub (See Availability of data and materials section).

From the set of 103 genome-wide significant DMRs called by DMRcate, there were several with substantive beta differences for exposure to preeclampsia and these spanned many adjacent CpG sites (Supplemental Table S2). In total, 62 of the 750 (8.3%) significant DMPs overlapped with these regions, and 47 of the 103 DMRs (45.6%) contained at least one genome-wide significant DMC (Supplemental Table S1).

DMR1 (*p* = 1.62 × 10^-42^; chr1: 45,189,335–45,191,110), the most statistically significant region, as weighted by Fisher’s multiple comparison statistic p-value, is 10 CpG sites and 1,776 bp across and spans exons 8 to 13 of the gene Armadillo-like Helical Domain Containing 1 (*ARMH1*; previously annotated as the open reading frame *C1orf228*). DMR2 is 1,574 bp and across a large span of 40 CpG sites (*p* = 9.35 × 10^-37^; chr20: 36,148,133–36,149,706), and bridging the promoter and exon 1 of the paternally imprinted neuronatin (*NNAT*), and exons and introns on the opposite strand of the isoform-dependent imprinted gene, bladder cancer associated protein (*BLCAP*) (Fig. 1a). DMR3 (*p* = 2.03E-31; chr22:31,001,766–31,003,655) is a large 1.9 kb and 19 CpG site DMR across the promoter and exon 1 of Pescadillo ribosomal biogenesis factor 1 (*PES1*) and transcobalamin (*TCN2*). The fourth-most statistically significant region, DMR4 (*p* = 7.33 × 10^-27^; chr6:33,047,944–33,049,360) is 1.4 kbp in length and has particularly large maximum (~ 0.13) and mean (~ 0.08) beta differences. It is placed across 12 CpG sites within the major histocompatibility complex (MHC) region of chromosome 6, spanning the human leukocyte antigen gene complex (HLA) alleles DP beta 1 (*HLA-DRB1*) and DP alpha 1 (*HLA-DRA1*) (Fig. 1b).

DMRs 5, 7, 9, 11, and 20 are all located within 6.7 Mbp from each other on chromosome 19. DMR5 spans the promoter and exon 1 of KLK7 (chr19:51,486,901–51,487,968) and 902 kb away is DMR11 across the promoter and exon 1 of ZNF577 (chr19:52,390,810–52,392,100). Around 1.1 Mbp upstream from DMR5 is DMR20 (chr19:50,391,064–50,391,885), with miRNA 4750 (*MIR4750*) centrally located and within the gene body of TBC1 Domain Family Member 17 (*TBC1D17)*. DMR7 (chr19:45,737,011–45,738,115) is only 16 kb away from DMR9 and it spans the promoter and exon 1 of Exocyst complex component 3-like 2 (*EXOC3L2*), while DMR9 (chr19:45,720,199–45,721,076) is proximal to Microtubule affinity regulating kinase 4 (*MARK4*) and across an exon of *EXOC3L2*.

For the remaining ten most significant DMRs, DMR6 (chr5:140,810,051–140,811,343) is a 16 CpG site 1.3 kb DMR lying across the promoter and exon 1 of protocadherin gamma subfamily A, 12 (*PCDHGA12*), DMR8 is (chr16:3,242,639–3,243,908) is within an intron of Olfactory Receptor Family 1 Subfamily F Member 1 (*OR1F1*), and DMR10 (chr2:109,746,251–109,747,264) is within exon 1 of SH3 Domain Containing Ring Finger 3 (*SH3RF3*). Beyond the ten-most significant DMRs, one other DMR has long runs of neighbouring differentially methylated probes with large differences, lending support to it being a genuine region of interest. DMR13 (chr16:1,583,391–1,584,516) is 12 CpG sites and 1.1 kb long with large maximum (~ 0.09) and mean (~ 0.06) beta differences. It spans the exons of Transmembrane protein 204 (*TMEM204*) and an intron of Intraflagellar transport 140 (*IFT140*).

We note that associations with DNA methylation may be simple or more complex. For example, DMR2 which spans across *BLCAP* and *NNAT* shows an unambiguous DNA methylation reduction in blood from preeclampsia-exposed children (Fig. 1a), whereas with DMR4, the region just downstream of the DMR has DNA methylation split into three clusters (Fig. 1b). This triclustered pattern is typical of methylation quantitative trait loci (mQTL) and could be arising from the common C to T (rs9277358) or G to A (rs3128961) polymorphisms which sit within the CpG sites on the BeadChip array (respective ids of cg20981163 and cg23333490). While there is a complex interplay between epigenetic and genetic regulation at this locus, the allelic methylation pattern is not associated with preeclampsia exposure (Fisher Exact *p* = 0.80).

### Comparison to existing studies

Existing EWAS for preeclampsia exposure make use of samples collected at birth. Most consider DNA methylation changes in placenta (with a meta-analysis in [56]), while two more recent larger studies have examined cord blood [18, 57]. Investigators from the Vitamin D Antenatal Asthma Reduction Trial (VDAART) trial examined cord blood DNA from 128 subjects (16 exposed to preeclampsia and 112 uncomplicated pregnancies). Covariates in the model included fetal sex, premature or term gestational age at delivery, and estimated cell type proportions. The Pregnancy and Childhood Epigenetics (PACE) consortium performed a preeclampsia meta-analysis across 3 cohorts (105 cases and 2,219 controls). They also undertook DMR calling with DMRcate. Models were run within each cohort, and the covariates included fetal sex, parity, estimated cell type proportions and maternal factors—age, smoking status, diabetes and pre-pregnancy BMI. We may compare this present study to these Illumina HumanMethylation450 (450 K) BeadChip based studies as 410 of the 750 probes significant in this EPIC BeadChip based analysis are present on the 450 K design (Table 4). Conversely, 247 of 263 CpG sites significant in the VDAART cohort are on the EPIC array, as are 494 of 542 for the fully-adjusted PACE consortium model and 7,735 from 8,252 for the unadjusted model.

**Table 4:** Comparison with cord blood differentially methylated probe findings

	Set sizes		VDAART—full model			PACE—full model			PACE—unadjusted model		
	Here	Common	Overlap	*p*-value	Odds ratio	Overlap	*p*-value	Odds ratio	Overlap	*p*-value	Odds ratio
n	865,859	452,453	247	–	–	494	–	–	7,735	–	–
*Probes filtered out on these thresholds*											
Detection p-value	5,373	2,333	1	1	0.65	0	0.082	0.00	18	1.42E-06	0.37
SNPs	41,560	22,649	4	0.001	0.26	30	0.925	1.01	306	9.50E-16	0.64
Non-autosomal	19,627	10,583	0	0.002	0.00	0	1.64E-06	0.00	0	2.10E-95	0.00
Batch-prone	14,394	7,886	1	0.071	0.19	10	1	0.96	53	2.75E-23	0.32
Cross-hybridising	29233	27,224	0	1.88E-08	0.00	0	1.56E-16	0.00	0	2.41E-250	0.00
Likely clustered	11,057	6,002	0	0.038	0.00	4	0.206	0.50	39	1.27E-18	0.31
*Significant probe overlap*											
Preeclampsia	750	410	0	1	0.00	0	1	0.00	5	0.295	0.59
CD4 + T	89,884	35,946	17	0.193	0.70	134	7.88E-29	3.52	3,176	< 2.2E-16	6.59
CD8 + T	48,602	18,382	4	0.011	0.32	76	1.88E-18	3.53	2,263	< 2.2E-16	8.04
B	32,359	12,261	3	0.072	0.36	31	0.001	1.99	1,386	< 2.2E-16	6.47
Natural killer	76,184	30,268	12	0.078	0.58	75	1.34E-07	2.04	2,136	< 2.2E-16	4.35
Eosinophils	18,313	6,794	3	0.636	0.67	12	0.306	1.35	385	6.60E-65	2.84
Any blood	175,881	73,683	28	7.12E-04	0.52	193	2.77E-23	2.63	4,016	< 2.2E-16	4.43

No CpG sites overlapped with the VDAART study or the PACE fully adjusted model. For the 7,735 shared significant CpGs in the PACE unadjusted model, a total of 5 probes overlapped with those significant here (cg04062715, cg13924580, cg01534390, cg17500686, cg26897081). However, if the likelihood of that overlap is considered, this is no more than expected by chance (Fisher Exact *p* = 0.295, odds ratio = 0.59) (Table 4). The PACE DMRcate analysis found 185 regions and there is an almost exact overlap of one DMR (chr19:52,390,810–52,391,090) with DMR11 here and positioned within the DMR hotspot on chr 19. The VDAART and PACE results also showed little correspondence with each other, with no CpG sites overlapping between the VDAART results and PACE fully adjusted model and two when comparing to the unadjusted model (cg12884009 and cg14459158).

To further investigate inter-study technical differences, an analysis was made comparing probes in this study which were filtered out or found to be associated with blood cell types (Table 4). The PACE study was more permissive in allowing CpG sites proximal to SNPs, but both the VDAART and PACE studies had little to no significant probe overlap with those considered here as non-autosomal, cross-hybridising, or unreliable (detection p-value and batch-prone) suggesting some concordance in probe-wise quality control measures with this present study. The analysis found that the PACE study, in particular, had many probes considered associated with preeclampsia exposure as associated in this current study with one or more blood cell types (Table 4). Compared with the PACE unadjusted model, the PACE fully adjusted model, which took into account blood cell types, had 39.1% (193 of 494) of significant probes overlapping with any blood cell type rather than 51.9% (4,016 of 7,735). The VDAART study had 11.3% (28 of 247) of probes overlapping with blood cell types. Both the VDAART and PACE consortium studies have far more overlap of their significant probes with blood cell types than associations with preeclampsia.

### DMR enrichment analysis

The set of 103 DMRs was examined for enrichment across GO terms, KEGG pathways and 50 Hallmark gene sets from MSigDB. A small set of 103 DMRs has very modest power to find enrichment as there can only be a maximum of 103 instances of sampling without replacement for a hypergeometric test. This leads to reduced ability to correctly reject a false null hypothesis and creates some dependencies upon the size of the biological category. Despite these constraints, five GO biological process terms and one molecular function term were found to be significant after BH-FDR correction of p-values (Table 5, bold denotes significance at an alpha of 0.05). Three GO biological process terms were child and parent terms in the ontology (GO:0007156, GO:0098609, GO:0007155). The terminal child term, GO:0007156 (homophilic cell adhesion via plasma membrane adhesion molecules), had 17 of 163 genes differentially methylated and was highly significant (*p* = 7.49 × 10^–12^). The biological process in GO:0007156 is defined as the attachment of a plasma membrane adhesion molecule in one cell to an identical molecule in an adjacent cell. This set is composed largely of cadherins, protocadherins, and cell adhesion molecules. The cadherins are calcium-dependent and we note the significant GO molecular function term (GO:0005509) is around calcium ion binding. In the KEGG pathway analysis, there were no terms significant after BH-FDR correction of p-values, but the top three terms (Table 5) are all immune related.

**Table 5:** DMR enrichment analysis

GO Identifier	Ontology and term	Set (n)	DMRs (n)	p-value	FDR* p*-value
GO:0007156	BP; homophilic cell adhesion via plasma membrane adhesion molecules	163	17	4.18E-16	**7.49E-12**
GO:0098742	BP; cell–cell adhesion via plasma-membrane adhesion molecules	269	20	6.77E-16	**7.49E-12**
GO:0098609	BP; cell–cell adhesion	952	23	4.42E-08	**3.26E-04**
GO:0005509	MF; calcium ion binding	676	18	3.74E-07	**2.07E-03**
GO:0007399	BP; nervous system development	2518	36	8.51E-06	**0.038**
GO:0007155	BP; cell adhesion	1489	25	1.25E-05	**0.046**
KEGG pathway	Description	Set (n)	DMRs (n)	*p*-value	FDR* p*-value
hsa05134	Legionellosis	56	3	0.004	0.669
hsa05145	Toxoplasmosis	106	4	0.005	0.669
hsa04612	Antigen processing and presentation	70	3	0.005	0.669

### Epigenetic clock analysis

The Horvath skin and blood epigenetic clock, implemented in the R library methylclock, was computed for all 40 samples. The checkClocks function of methylclock reported no CpG sites were missing in the calculation. The DNA methylation age residuals subtracted from chronological age had two measures computed; extrinsic epigenetic age acceleration (EEAA), which takes into account age-related functional decline of the immune system such as decreases in naïve CD8 + T cells and increases in memory or exhausted CD8 + T cells and, intrinsic epigenetic age acceleration (IEAA), which adjusts by cell-type to measure age acceleration independently of age-related changes in the cellular composition of blood. Epigenetic age was estimated on data normalised by noob and without batch effect correction.

**Fig. 2:**
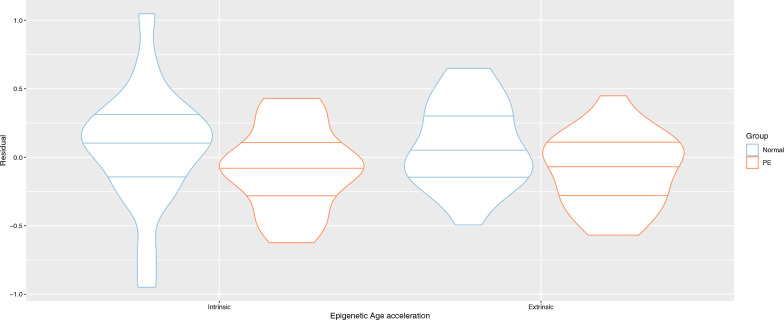
Epigenetic age acceleration in offspring of preeclampsia versus normotensive pregnancy. Intrinsic and Extrinsic epigenetic age acceleration (y-axis, measured in years) was reduced for children exposed to intrauterine preeclampsia (orange) compared to normotensive pregnancies (blue), however these differences were not nominally significant

In the children exposed to preeclampsia, there was a consistent small reduction in epigenetic age across both the EEAA and IEAA measures, with a difference between the means of the residuals of 0.1836 and 0.1515 years, respectively (Fig. 2). The residuals were found to be sufficiently normally distributed (insignificant Shapiro–Wilk normality test), so a Welch Two Sample t-test was used to compare the groups. Neither the EEAA measure (*p* = 0.057) nor IEAA measure (*p* = 0.215) were nominally significantly different, but the EEAA result was suggestive.

### Telomere analysis

**Fig. 3:**
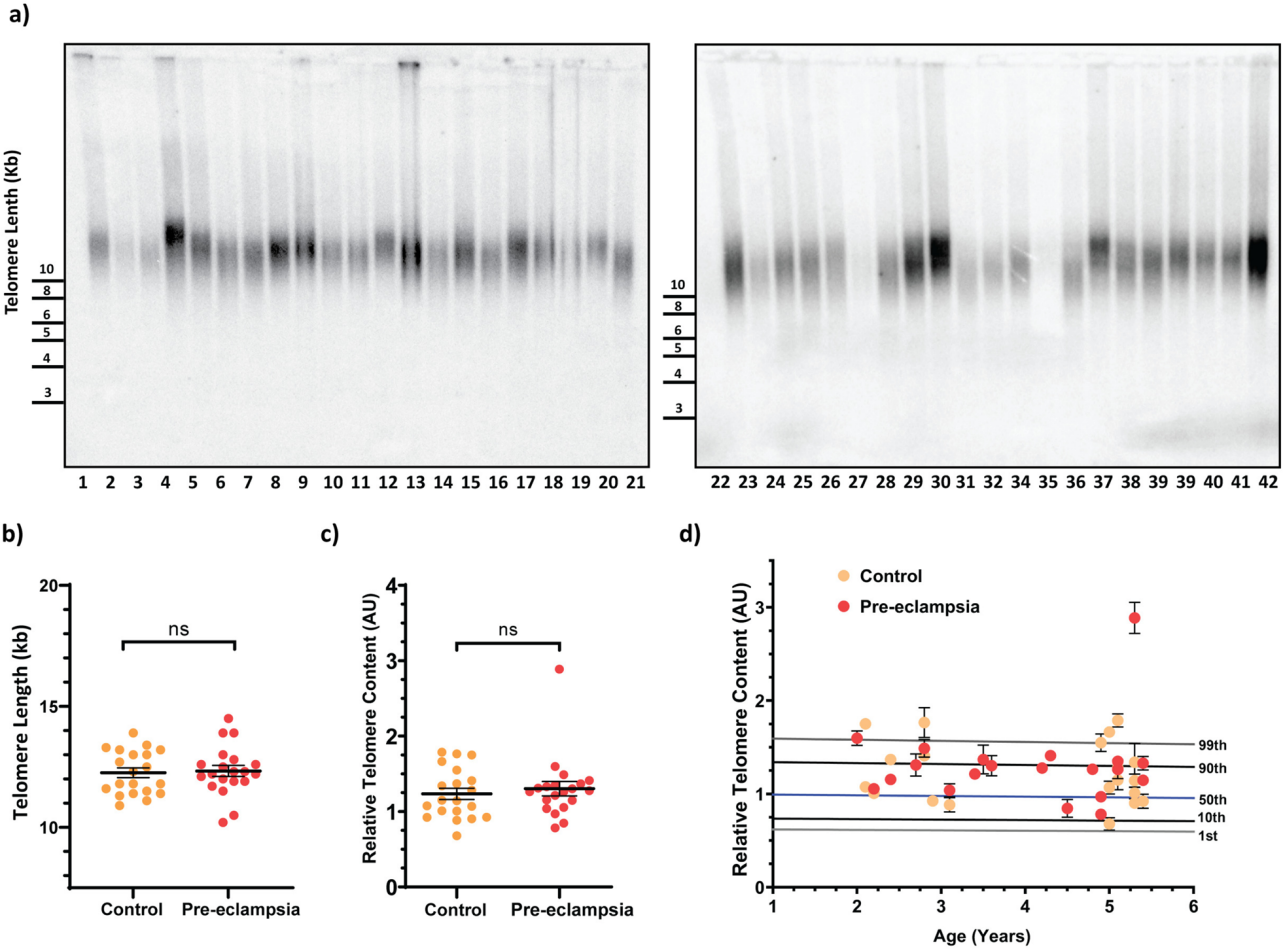
Telomere length analysis in offspring of preeclampsia versus normotensive pregnancy. **a** Terminal Restriction Fragment (TRF) length analysis across the cohort. **b** Quantitation of TRF lengths (kb) using ImageQuant TL analysis software (2018, Cytiva) for control (orange) and pre-eclampsia (red) groups. Statistics performed using Student’s t-test. **c** Relative telomere content (ratio between telomere and HBG single copy gene cycle thresholds) analysed by qPCR for control (orange) and pre-eclampsia (red) groups. Statistics performed using Student’s t-test. **d** Relative telomere content analysed by qPCR plotted against age across the cohort. Lines indicate the 1st, 10th, 50th, 90th, and 99th telomere content percentiles from a cohort of 240 normal controls

To measure telomere length across the cohort, we employed two complementary techniques; terminal restriction fragment (TRF) length analysis (Fig. 3a and b), which involves restriction enzyme digestion, gel electrophoresis, and hybridisation to a radioactively-labelled telomeric probe, and quantitative PCR (qPCR) (Fig. 3c and d), which uses PCR amplification of telomeric repeats compared to a single copy gene to measure relative telomere content. Telomere length measurement by the two techniques was comparable (Fig. 3a to d), with one notable outlier in the preeclampsia group, which presented with very long telomeres, as detected by both TRF and qPCR analysis (sample 5). No significant differences in telomere length were detected between children exposed to intrauterine preeclampsia and the control group using either telomere length measurement technique (Fig. 3b and c).

## Discussion

In this study, we found that exposure to preeclampsia is associated with 750 differentially methylated probes (DMPs) and 103 differentially methylated regions (DMRs) in the blood of young children. This is the first study to consider such epigenetic associations in relation to preeclampsia exposure, with previous epigenome-wide studies focused on DNA methylation differences in maternal blood, cord blood, or placental biopsies. Principle components analysis revealed the influence on the methylation data of variable leukocyte proportions across children and the importance of accounting for cell types in association testing. Presumably, much of the difference in leukocyte proportions is due to age [58], or the coincidence of recent respiratory infections or allergies in these young children [59].

Metanalyses exist in the literature, and a re-analysis of seven placenta-focused datasets by Almomani et al. in 2021 [56] found little consistency in DMPs between each study. The authors suggest this lack of reproducibility could be due to small study sizes, the heterogeneity of preeclampsia, and the lack of characterising cell-specific methylation profiles in the placenta. The capacity to undertake placenta cell type proportion analysis requires reference sets and these have only appeared in the literature more recently [60, 61]. Studies based on cord blood may use blood-based cell type references as the resources to undertake this have a much longer history [62]. Both the VDAART and PACE studies incorporate correction for blood cell type proportions similar to this current analysis, and the present results were compared.

In alignment with earlier findings in placenta, there was very little reproducibility of results between this present study and the VDAART or PACE work, or between VDAART and PACE. While the VDAART and PACE consortium studies do account for cell type proportions in their fully-adjusted models, many of the DMPs they reported as associated with preeclampsia exposure are found to be associated here with blood cell type. When comparing the two PACE analyses, the large difference in p-values and odds ratios for the blood cell types between the corrected and uncorrected PACE models demonstrates a favourable reduction in false positive results due to incorporating cell type into the model. However, the results here suggest this was not sufficient. The power to remove blood-cell type false positive results in the PACE study is hampered by the meta-EWAS design, with each cohort undertaking blood cell type estimation separately and with variable batch-effect correction procedures. The use here of a fully-adjusted model in the batch effect correction preserves cell type variation while reducing technical variation and yields more power to detect DMPs associated with blood type [38].

A recent preprint from Yang et al. reanalysed the PACE consortium data as well as several other cord blood and placenta focused studies [63]. They found that many previously reported DMPs associated with severe preeclampsia are artifacts caused by confounding factors such as cell type heterogeneity and gestational age. They also note that gestational age has an impact on the proportions of blood cell types and remark that studies of cord blood need to include this factor into their models.

To reduce false positive results, the analysis here focused on DMRs, which relies upon DMPs to be clustered and have neighbouring support. In certain circumstances, batch-effect correction can also introduce false positive DMPs [52]. To further understand the underlying biology involved in these DMRs, we performed enrichment analysis. The results implicated epigenetic changes in homophilic cell adhesion. An earlier study which examined the DNA methylation profiles in preeclampsia and healthy control placentas discovered the same GO term (GO:0007156) as the topmost enriched biological process [64]. An association with differential methylation of cadherin and cell adhesion molecules was also noted in an earlier genome-wide DNA methylation study of preterm preeclampsia placentas [65]. Existing work studying placental tissue, and this current work on blood from preschool children, consistently implicate epigenetic changes in homophilic cell adhesion. Interestingly, abnormal expression of cell adhesion molecules by cytotrophoblasts during pregnancy [66] is also associated with preeclampsia.

The DMR analysis highlighted several genes which are known or likely biomarkers for preeclampsia. DMR2 spans Bladder cancer-associated protein (BLCAP), a known differentially methylated gene in the placenta associated with preeclampsia [67], and the maternally imprinted Neuronatin (*NNAT*) locus has raised expression in the severely preeclamptic placenta [68]. DMR40 spans the Angiopoietin-2 (*ANGPT2*) locus and this gene product is an antagonist of angiopoietin-1 which disrupts vascular remodelling ability. Angiopoietin-2 is a known biomarker for preeclampsia with the placental expression of angiopoietin-2 found to be significantly increased in preeclamptic patients [69, 70]. Endothelial cells in developing blood vessels express elevated transcript levels of *EXOC3L2* (DMR7, DMR9) [71]. Transcobalamin (TCN2) transports cobalamin (vitamin B12) from the bloodstream, and a recent meta-analysis showed that women with preeclampsia had significantly lower cobalamin concentrations than normotensive pregnant women (DMR3) [72].

This present study cannot elucidate between epigenetic adaptation and genetic inheritance that is observable via an epigenetic signature. For example, DMR4, which has a triclustered methylation pattern directly downstream and known common C to T (rs9277358) or G to A (rs3128961) polymorphisms sitting within CpG sites in the DMR has the hallmarks of a *cis*-methylation QTL. This DMR spans the *HLA-DPB1*/*HLA-DPA1* locus within the MHC region, which is a dense hotspot for correlated regions of systemic interindividual variation in DNA methylation (CoRSIVs). The majority of these CoRSIVs are mQTLs [73]. The reduced methylation in children from preeclamptic pregnancies could be solely due to prenatal exposure to preeclampsia (epigenetically driven) but may also reflect the inheritance of risk factor allele(s) for preeclampsia that influence methylation (methylation QTL). In the methylation QTL instance, the observed change in methylation is a proxy and latent variable for inherited Human Leukocyte Antigen (HLA) risk alleles. Support for this notion comes from causality work in multiple sclerosis (MS). Causal inference testing has shown that DNA methylation directly mediates the relationship between MHC region genetic variants and MS [74]. In another study, causal inference testing and Mendelian randomisation demonstrated that HLA genetic variants mediate risk for MS via methylation changes in a DMR within *HLA-DRB1*, leading to changes in HLA-DRB1 expression [75]. Alternatively, it is also possible that differential methylation at this locus may be independent of genetics and additive in effect. Further elucidation of the interplay between the genetics and epigenetics is required, particularly for the highly polymorphic major histocompatibility complex (MHC) genes of the adaptive immune system.

There are further potentially confounded examples where both genetic inheritance and environmental exposure can explain the observed difference in methylation. One example is DMR3, which spans transcobalamin. The observed altered methylation could be due to a maternally inherited allele associated with reduced maternal cobalamin adsorption (a methylation QTL example). Reduced circulating maternal cobalamin concentration during pregnancy is an established risk factor for preeclampsia [72]. Alternatively, the altered methylation may be an adaptation by the fetus to an in utero environment with low concentrations of circulating cobalamin. In a further example, we speculate that DMR29 (proximal to *RPH3AL*) may not be caused directly by exposure to preeclampsia in utero, but could just be correlated. Differential methylation of *RPH3AL* is associated with IgE food allergy at 12 months of age [76] and it is known that preeclampsia associates with food allergy and an increased amount of total IgE in childhood [77]. The possibility exists that exposure to preeclampsia leads to conditions that cause *RPH3AL* methylation later during infanthood.

This study also considered differences in leukocyte telomere length and a DNA methylation-based epigenetic clock. Telomere length and epigenetic clocks are only modestly correlated with each other [78]. No significant differences in telomere length were observed in the children exposed to intrauterine preeclampsia compared to the control group. The use of two complementary telomere length measurement techniques strengthens this finding and is indicative of preeclampsia not having a significant impact on early-age offspring telomere length. It is, however, possible that the 2–5 year offspring age window is limiting, and that telomere length factors may present at more advanced ages, consistent with adverse long-term health outcomes [79].

In interpreting molecular aging data, we note that exposure to preeclampsia is confounded by other clinical variables, including gestational age at delivery. In the context of other findings, most of the literature focuses on telomere lengths at birth. However, in children, leukocyte telomere length at the age of 4 correlates with telomere length in neonate cord blood (r = 0.71) and placenta (r = 0.60) [80].

Previously, leukocyte telomere length in blood from newborns small for gestational age was found to be longer than telomeres in large for gestational age newborns [81]. In this current study, we note birth weight Z-scores corrected for gestational age were not different between the preeclampsia and normotensive pregnancy groups.

Epigenetic age was found to be marginally decelerated in preeclampsia-exposed children, but not significantly so. This decrease is consistent with a recent large preeclampsia study (57 preeclampsia cases, 1401 controls) examining a gestational age epigenetic clock in cord blood and newborn blood spots [82].

This present study highlights known biomarkers and new potential candidate genes by examining DNA methylation and biological ageing for the first time in preschool aged children. However, our sample size was limited, highlighting the need for a larger study which encompasses both inspection of DNA methylation via an epigenome-wide association study, and genotyping via a genome-wide association study. This would enable separation of the two confounded mechanisms affecting the interpretation of our current study’s findings; 1) genetic inheritance (observable as methylation QTL) and, 2) epigenetic adaptations made by the blood stem cells due to in utero environmental exposure. In future studies, pan-tissue and longitudinal aspects would also establish which epigenetic changes are present in the cord blood or placenta, and which arise later and are observable changes in the blood of children.

## Conclusion

Telomere lengths and epigenetic age were similar between young children exposed to either preeclampsia or a normotensive pregnancy, indicative of no significant impact of preeclampsia exposure to early-age offspring. However, we did identify 103 differentially methylated regions in the blood of those exposed to preeclampsia, compared to children exposed to a normotensive pregnancy. Several regions identified support findings from previous research in placental tissue of women with preeclampsia, suggesting the transmission and persistence of DNA methylation to offspring. More research is needed to separate out confounding inheritable factors and understand the role of these differentially methylated sites in mediating the effects of preeclampsia on short- and long-term health outcomes. Clinical application of these findings could contribute to the identification of environmental triggers for adult-onset disease and novel therapeutic targets.

## Supplementary Information

Supplementary material 1

Supplementary material 2

## Data Availability

R code for this analysis is available at Github (https://github.com/JasonR055/P4PSS_Preeclampsia.git). Raw MethylationEPIC IDAT data has been deposited at the CSIRO Data Access Portal (10.25919/6wzx-0098). The approved ethics for this data does not allow for public release, instead interested researchers should use the contact email address given in the Data Access Portal.
