# Supplementary figures and images for: DNA methylation and telomere length in 2–5 year olds with intrauterine preeclampsia exposure: a P4 sub-study
Source: Clin Epigenetics. 2025 Nov 24;18:1. doi: 10.1186/s13148-025-02029-1 (PMC12763936; doi:10.1186/s13148-025-02029-1)

**Gender**

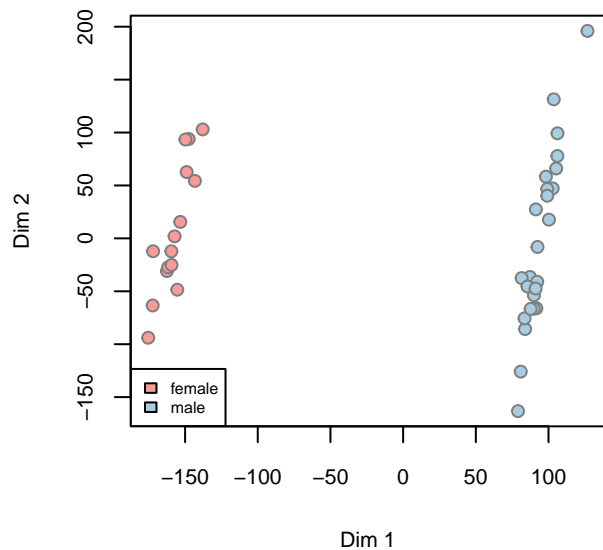

**Neutrophil proportion**

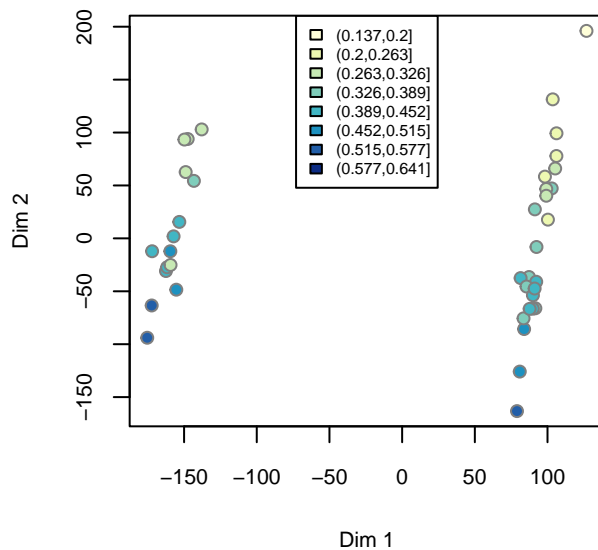

**Age (yrs)**

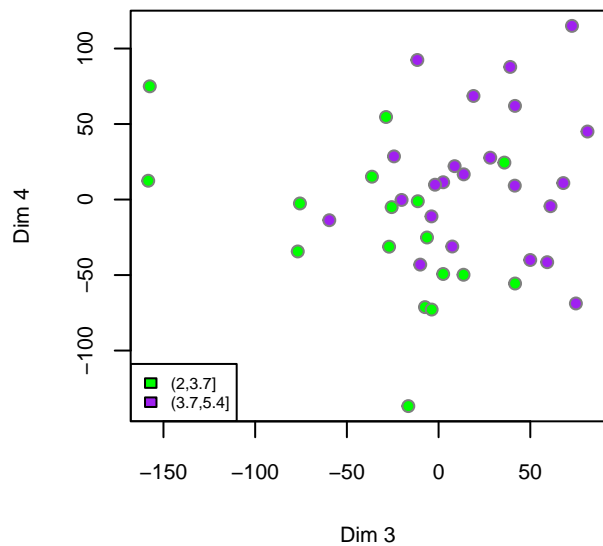

**Slide identifier**

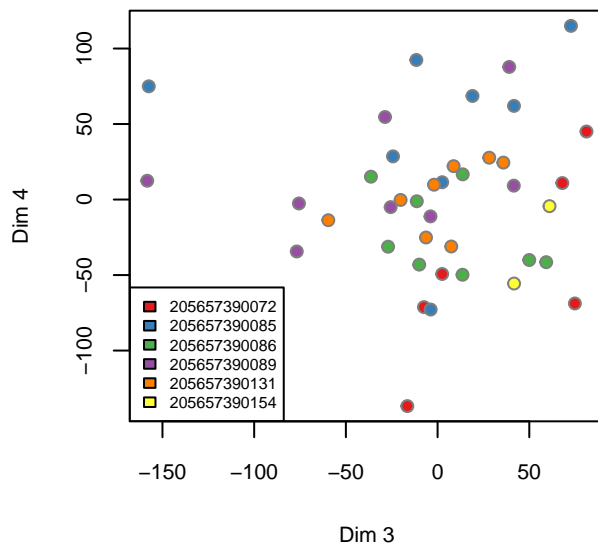

Slide identifier

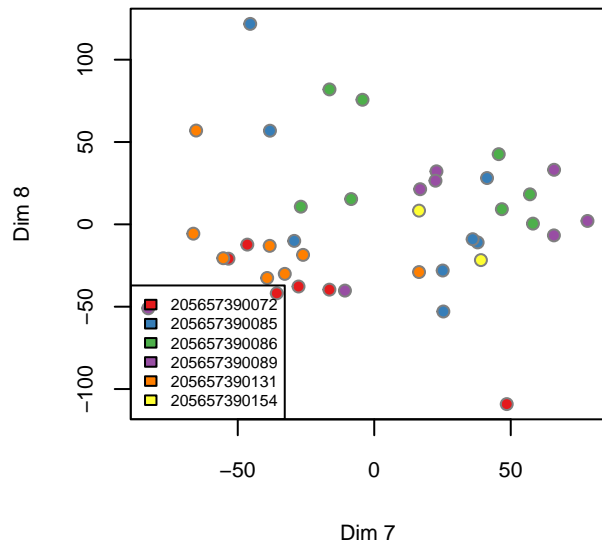

Slide identifier

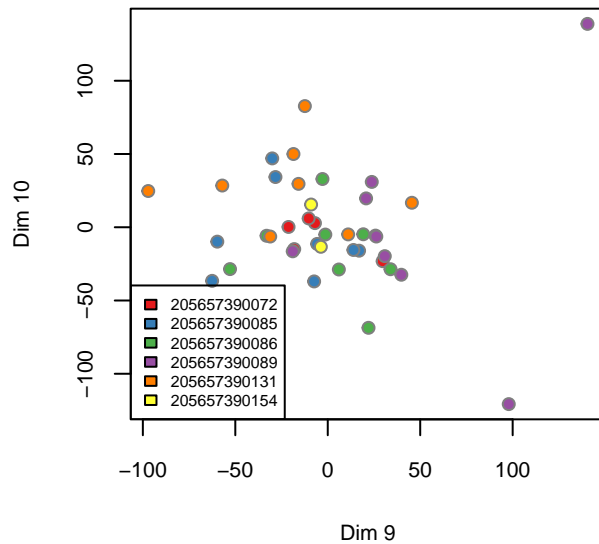

Supplement: Supplementary file 2 — Supplementary material 2 [file 13148_2025_2029_MOESM2_ESM.pdf]
